# Revolutionizing agroecosystems through next-generation bio-nanofertilizers: an overview toward sustainable agriculture

**DOI:** 10.1039/d6na00006a

**Published:** 2026-02-24

**Authors:** Samukarani Swain, Lala Behari Sukla, D. P. Krishna Samal

**Affiliations:** a Biofuels and Bioprocessing Research Centre, Institute of Technical Education and Research, Siksha ‘O’ Anusandhan (Deemed to be University) Bhubaneswar-751030 India lalabeharisukla@soa.ac.in

## Abstract

The rising global population demands a 70% increase in crop production to satisfy future food needs. This challenge requires a collaborative effort to boost food production while maintaining sustainable agricultural practices. While common chemical fertilizers can promote plant growth and yield, their overuse damages soil biota and fertility and pollutes the environment. Therefore, nanofertilizers are emerging as a promising alternative to conventional options, helping to reduce environmental impact and becoming essential for future farming. Bionanofertilizers are advanced materials made of nanoscale particles and beneficial microbes engineered to supply vital macro- and micronutrients. They improve nutrient efficiency and lessen environmental harm. These fertilizers provide controlled nutrient release, targeted delivery, and better bioavailability, overcoming the limitations of traditional fertilizers such as nutrient loss, soil degradation, and pollution. They promote plant growth, photosynthesis, and stress resistance, while also enhancing soil health and decreasing application frequency and costs. The need for nanofertilizers is 30 to 100 times lower than that for chemical fertilizers, making them more economical. On average, they increase yields by 24 to 32% across various crops and soil types. This review assesses evidence suggesting that bionanofertilizers can transform agricultural practices and provide sustainable, eco-friendly solutions for enhancing soil health and crop management.

## Abstract 2

Synthesis of bio-nano fertilizers and their application in sustainable agriculture.

## Graphical abstract

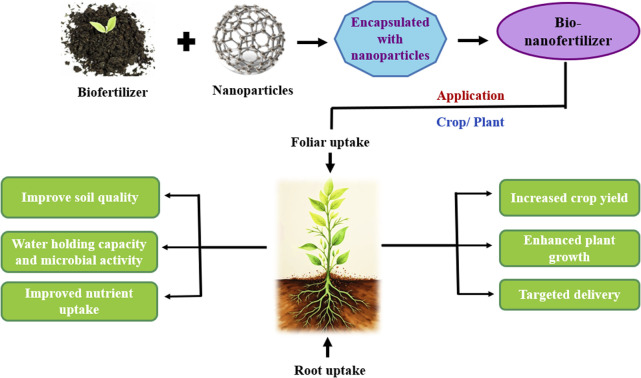

## 1 Introduction

Global population growth has driven an extraordinary surge in food demand, necessitating agriculture to deliver ever-higher crop yields. Since 1960, as the world's population has soared past 8 billion, crop production and chemical fertilizer use have risen sharply to keep pace. The demand for fertilizers has consistently outpaced growth in population and crop production, underscoring their key role in food security. With an estimated global population of nearly 10 billion by 2050, the pressure to maximize yields on limited arable land will only intensify.^1^ Traditional fertilizers have been essential in increasing crop production; nonetheless, their extensive and intensive application has caused notable environmental and health issues, such as soil degradation, water contamination and eutrophication, greenhouse gas emissions, air pollution, loss of biodiversity, and heavy metal pollution. Continuous use of chemical fertilizers depletes essential soil organic matter and disrupts microbial communities, leading to soil acidification, reduced fertility, and diminished water and nutrient retention.^2^

These mounting challenges have catalyzed a shift toward more sustainable solutions. Nanotechnology offers a transformative approach by enabling the development of bionanofertilizers. Bionanofertilizers combine nanotechnology with beneficial microorganisms (like bacteria/fungi) and nutrients, creating advanced, eco-friendly products that significantly boost plant growth, nutrient uptake and stress tolerance while reducing chemical fertilizer use.^3^ Bionanofertilizers are an emerging technology in agriculture that involves nanoparticles to enhance nutrient delivery to plants. Bionanoparticles, small molecules ranging from 1 to 100 nm, possess various properties and are used in several fields, including agriculture. Key features of nanofertilizers help resolve nutrient shortages, making them a valuable tool in various sectors.^4^ Bionanofertilizers deliver nutrients in a controlled, targeted manner, enhancing plant uptake, reducing nutrient losses and supporting soil microbial health. This innovation not only improves crop yields and nutrient use efficiency but also minimizes environmental harm, aligning with the goals of sustainable agriculture. This reduces nutrient waste and enhances uptake efficiency by up to 90% compared to chemical fertilizers.^5^ Furthermore, it can enhance plant resilience to abiotic stresses (*e.g.*, drought and salinity) by increasing enzymatic activity and osmolyte production, resulting in better survival and biomass growth.

This review examines the potential of bionanofertilizers in advancing sustainable agriculture, highlighting their role in enhancing crop productivity and improving soil fertility. It assesses their potential in reducing dependency on chemical fertilizers and minimizing environmental hazards. It discusses the contributions towards sustainable agricultural practices and eco-friendly farming systems. It explores the mechanisms through which bionanofertilizers improve nutrient uptake efficiency, along with research gaps and regulatory development to ensure their safe and effective adoption for large-scale application in agroecosystems.

## 2 Biofertilizers

Biofertilizers, composed of live microorganisms like bacteria, fungi, and algae, enhance soil fertility and plant growth health by fixing nitrogen, degrading phosphorus and transporting other nutrients. Biofertilizers contain beneficial microbes like blue-green algae (BGA), fungal mycorrhizae and plant growth-promoting rhizobacteria (PGPR), which enhance plant yield. The term “biofertilizer” originated from ancient Roman and Greek agricultural practices. By the late 19th century, bacteria such as *Rhizobium* and nitrogen-fixing microorganisms like *Azotobacter* and *Azospirillum* were recognized as effective biofertilizers. These organisms improve nutrient availability *via* biological nitrogen fixation (BNF) and solubilization of complex compounds, either as endosymbionts or in the rhizosphere.^6^ Biofertilizers enhance soil water retention, increase availability of key nutrients (N and P), and promote microbial growth, leading to improved aeration and natural fertilization processes.^7^ They also support fertilization by supplying nitrogen-fixing bacteria like *Frankia* and *Trichodesmium*. Through biological nitrogen fixation, these bacteria convert nitrogen gas into biologically available nitrogen that microorganisms can use. Simultaneously, dissolved minerals containing nutrients become more accessible for plant absorption.^8^ Biofertilizers are effective and environmentally friendly and sustainable since their application stimulates plant growth, improves nutrient availability and synthesizes phytohormones.^9^ However, regular biofertilizers have some limitations (Table 1). They don't last long on shelves, are sensitive to the environment, and are unstable in fields.^10^ To fix these issues, scientists have recently developed bionanofertilizers by mixing biofertilizers with nanometals. This approach uses nanotechnology to deliver nutrients to plants more efficiently and boost crop growth.

**Table 1:** Nanofertilizers *versus* biofertilizers

Features	Nanofertilizer	Biofertilizer	Citations
Nutrient release	Slow, controlled, and matches the plant's needs	Rapid, which often exceeds plant needs	11
Nutrient use efficiency	High	Lower	12
Uptake efficiency	3× higher due to nanoscale mobility	Low (20–30% absorption)	13
Soil health	Enriched microbes improve fertility	Can degrade soil quality	14
Microbial survival	Protected by coatings	Vulnerable to environmental stress	15
Environmental impact	Minimal pollution and eco-friendly	High risk of runoff and pollution	16
Crop yield and quality	Improved yield and stress tolerance	May boost yield and quality varies	17
Cost and resource use	Lower input is needed and cost-effective	Higher input and costs	18
Disease resistance	Enhanced vis bioactive compounds	Limited and may require pesticides	12

## 3 Bionanofertilizers

Bionanofertilizers combine the benefits of nanotechnology and biofertilization to enhance nutrient availability and uptake by plants. They work by pairing microbes with nanoparticles for controlled, efficient delivery, improving nutrient use efficiency, enhancing soil health and protecting crops from pathogens, paving the way for sustainable agriculture.^19^ Nanoparticles, with sizes ranging from 1 to 100 nm, commonly include zinc, silicon, iron, copper and silver in nanofertilizer applications. Their absorption and accumulation in plants depend on chemical composition, shape, size and aggregation state, with larger nanoparticles exhibiting lower translocation.^20^ One method called nanoencapsulation protects the ingredients, helps them last longer, and prevents microbes that assist plants in growing in a controlled way.^21^ The subsequent formulation of non-encapsulated fertilizers improves the efficacy of fertilizers in agriculture by utilizing encapsulation in a polymer matrix, which prevents the loss of fertilisers and/or degradation in situations that pose a risk to the efficiency of the fertilisers. These fertilizers, available in nanopowder, nanocapsules and nanoemulsions, provide nutrients to plants, enhancing their productivity and quality.

**Fig. 1:**
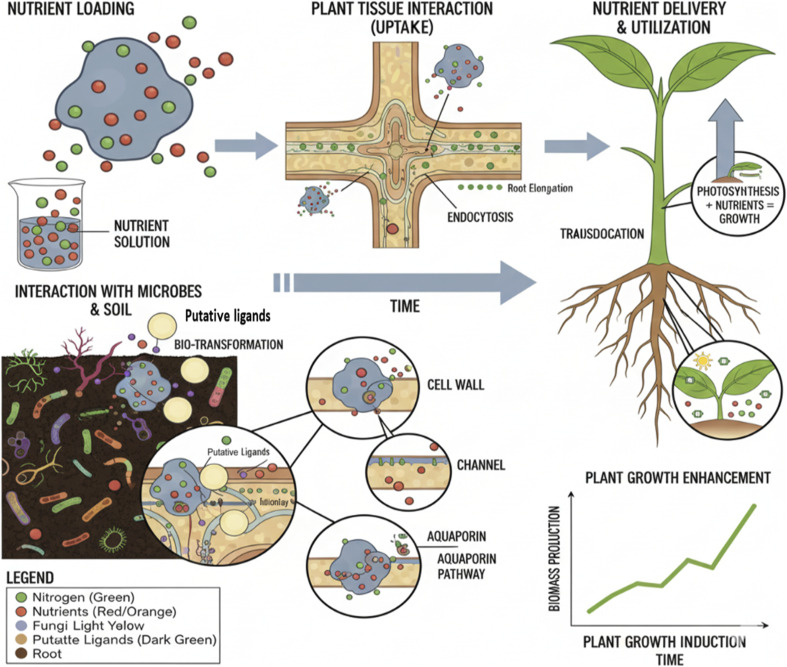
Mechanism of bionanofertilizer function (https://BioRender.com).

Bionanofertilizers offer several benefits. They release nutrients steadily, which helps plants absorb more nutrients. They work better in the field. They are cost-effective and eco-friendly. Bionanofertilizers fit into modern applications of nanotechnology in agriculture, directed toward the promotion of crop production.^22^ The high surface area to volume ratio of the nanoparticles allows better ground affinity with the plant surface and also enables the roots to increase nutrient uptake by plants by reducing the loss of nutrients through leaching, volatilization and runoff.^23^ The application of bionanofertilizers contributes to increased enzyme activity, promotes beneficial microbial populations, improves crop quality and enhances resistance to diseases.^24^ The potential advantages of bionanofertilizers are enhanced agricultural productivity, sustainability and food security. Additionally, plant species influence nanoparticle receptors, allowing a plant to act as both an accumulator and excluder of different nanoparticles. Nanoparticles enhance plant development through various strategies and are delivered *via* endocytosis, carrier proteins, or foliar sprays, moving through apoplastic and symplastic channels (Fig. 1).^25^

## 4 Bionanofertilizers improve soil fertility and crop yield

Traditional fertilizers have low uptake efficiencies and undergo rapid chemical transformations, which can harm both the soil and the environment. They preserve soil fertility and boost crop yields and can be applied directly or through foliar application. Nonetheless, the impact of bionanofertilizers can be influenced by organic and inorganic components of soil, based on their composition and interactions with the soil.^26^ Aggregation occurs right after the application of bionanofertilizers, reducing their effective action area and mobility. The quantity of organic materials in the soil and environment, along with the chemical properties of bionanofertilizers, can enhance or diminish their mobility.^16^

For instance, adding ZnO nanoparticles to zinc-deficient soil increased barley (*Hordeum vulgare* L.) yield by 91% and enhanced the efficiency of nutrient utilization.^27^ Nano-composite fertilizers enhance rhizosphere microorganisms, thereby fostering plant growth by promoting root surface colonisation.^27^ Controlled-release fertilizers aid in minimizing nitrogen leaching and runoff while enhancing wheat yield and elevating soil residual mineral nitrogen levels. The nanomaterials employed consist of silver, gold, aluminum, single- or multiwalled nanotubes, magnetized iron nanoparticles, zinc, zinc oxide, copper, silica, titanium dioxide, and cerium oxide. Silicon nano-biofertilizers reduce heavy metal uptake and UV stress in crops.^28^ They promote seed germination and photosynthesis, as nanoparticles like TiO_2_ and carbon nanotubes stimulate seed germination rates and enhance photosynthetic efficiency by up to 30%, directly increasing yield.^29^ They reduce environmental damage as conventional fertilizers contribute to eutrophication, greenhouse gas emissions, and soil degradation. Bionanofertilizers mitigate these issues by lowering chemical runoff as nano-capsulation reduces nutrient leaching by 40–70%, preventing water pollution and algal blooms; for instance, zinc oxide nanoparticles decrease phosphorus runoff in barley crops.^30^ Phosphate ions bind with the positive charge of the ZnO surface forming stable ZnP complexes and enabling rhizosphere retention by microbes that facilitate absorption and limit the mobility of phosphorus in barley. Eco-friendly composition by combining biofertilizers (*e.g.*, *Rhizobium* and *Pseudomonas*) with nanomaterials minimizes reliance on synthetic chemicals, preserving soil microbiota and reducing toxicity. Carbon footprint reduction is achieved as nanofertilizers are required in smaller quantities (20–50% less) due to higher efficiency, lowering transportation and application emissions.^31^ Due to lower inputs of bio-nanofertilizers there is a direct decrease in energy consumption associated with chemical fertilizer production. In addition they also reduce greenhouse gas emission by minimizing excess chemical fertilizer applications. Bionanofertilizers optimize improved nutrient efficiency management through a high-surface-area-to-volume ratio, as nanoparticles provide broader interaction surfaces, ensuring faster absorption and reduced nutrient loss. They promote synergistic microbial activities as biofertilizers paired with nanomaterials enhance nitrogen fixation and phosphate solubilization. For example, nanobiofertilizers with *Bacillus* spp. increase phosphorus availability by 60%. Precision delivery of nutrients is achieved in response to plant needs, avoiding over-fertilization.^32^ Studies show that nanofertilizers improve nitrogen use efficiency by 70% in wheat and rice.^32^ Economic and sustainability benefits like cost-effectiveness, reduced application frequency and quantity and lower farmer expenses by 30–50% are realized. As bio-nano fertilizers release nutrients in a targeted manner, more of the nutrients are absorbed by the plants. Hence fewer applications are required for more yield that directly lowers the cost of fertilizer purchase and application labour. Nano-biofertilizers enrich organic matter and microbial diversity, improving long-term fertility and soil health. Scalable green synthesis methods using plant extracts or microbes make production sustainable and renewable.^33^

**Fig. 2:**
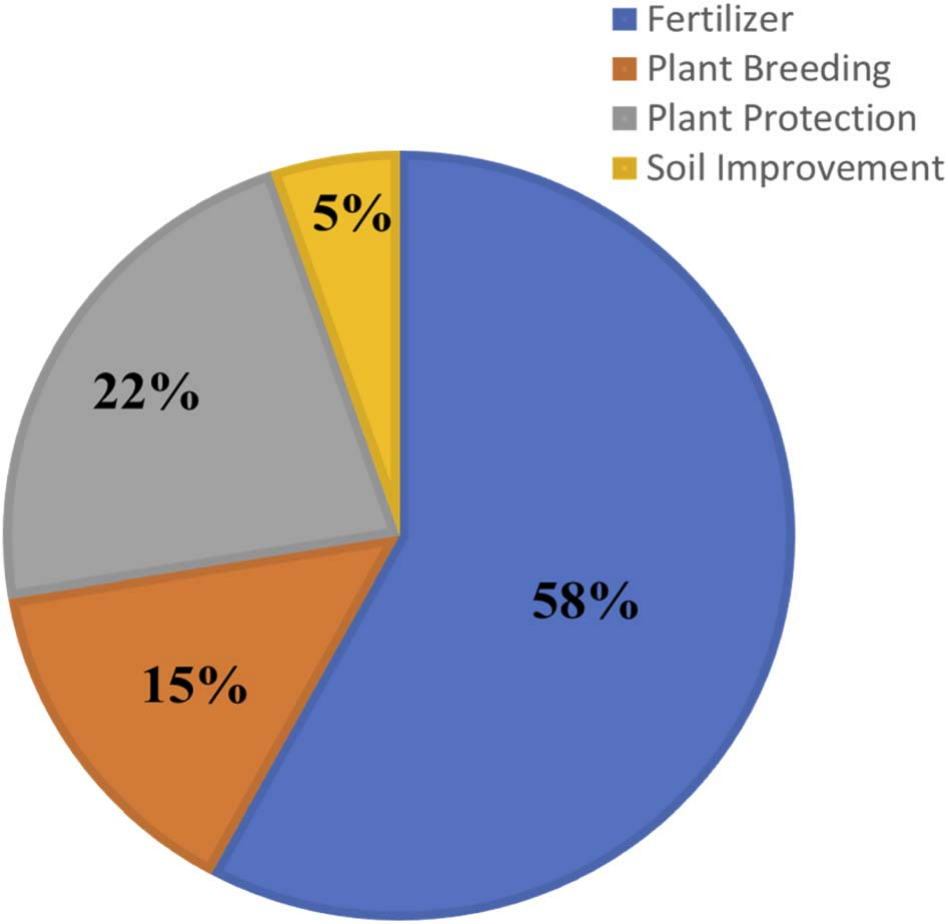
Agronomic product divisions based on the nanotechnology product database.

People are improving farming methods to boost food crop production and other agricultural goods. However, challenges like limited land, water shortages, climate change, pests and low efficiency hinder food security, while the rising global population increases the demand for fertilizers. Fertilizers are essential for plant growth and development, with ideal crop nutrition crucial for crop production. The yield of crops relies heavily on the presence of macronutrients (N, P, K, S, Ca, and Mg) and micronutrients (B, Fe, Mn, Cu, Zn, Mo and Cl) in agricultural lands.^34^ The world consumption of nitrogen, phosphorus and potassium increased by 1.5% from 2023 to 2024, with demand for these nutrients growing annually by 1.5%, 2.2% and 2.4%, respectively, from 2020 to 2024 and expected to continue increasing in the next four years.^35^ Agronomic product divisions based on nanotechnology products used in fertilizers, plant breeding, plant protection and soil improvement are presented (Fig. 2).

ZnO nanoparticles are essential micronutrients that enhance plant growth, yield and photosynthetic efficiency in crops like wheat and maize. Bionanofertilizers with nano-hydroxyapatite (nHAP) formulations of calcium phosphate nanoparticles are used as slow-release phosphorus fertilizers, improving nutrient use efficiency and promoting growth in various plants. Carbon nanotubes (CNTs) and graphene are carbon-based materials that can act as carriers for NPK and other micronutrients, improving their absorption by plant roots and enhancing soil water retention.^36^ Copper nanoparticles (Cu NPs) are effective against diseases like late blight in tomatoes, often outperforming traditional copper-based chemicals at lower application rates.^37^ Gold (Au) NPs and carbon nanotubes (CNTs) can serve as carriers to introduce the genetic material, such as specific RNAs or the components of the CRISPR/Cas9 system, into plant cells to improve traits like disease resistance or nutrient uptake efficiency. Multi-walled carbon nanotubes (MWCNTs) can penetrate the thick seed coat of various crops (*e.g.*, corn and soybeans) to enhance water absorption, leading to improved and more rapid seed germination and seedling growth.^38^ Nano-clays and zeolites help enhance the soil's water-holding capacity, nutrient retention, and aeration properties. This is particularly beneficial in drought-prone or poor-quality soils. Iron oxide (Fe_3_O_4_) NPs can help remediate heavy metal contamination in the soil by acting as adsorbents and they can also improve iron availability for plants in alkaline soils.^39^

Conventional fertilizers are inefficient and damage the environment. There is a need for higher crop yields with less environmental impact, better nutrient delivery, enhanced plant growth, stress resistance, lower costs, and smaller, customizable solutions for nutrition and protection. Nanofertilizers can address these issues by increasing nutrient efficiency, supporting sustainable crop production, and minimizing environmental harm. They represent a key innovation for sustainably ensuring future food security.^40^

## 5 Synthesis of bionanofertilizers

**Fig. 3:**
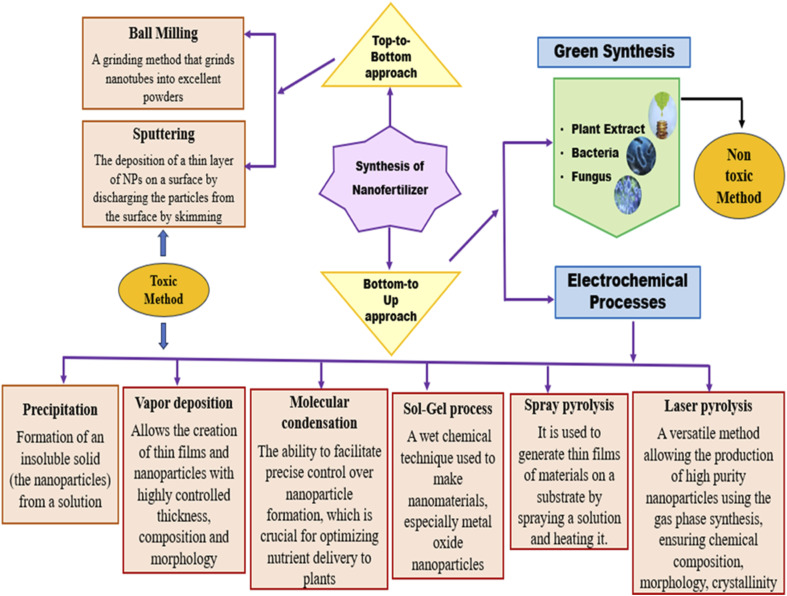
Different types of synthesis of bio-nanofertilizers.

Bionanofertilizers can be synthesized through various methodologies, including top–down and bottom–up approaches, which enable the controlled inclusion of necessary nutrients.^41^ Mass production of nanoparticles involves the synthesis of nanoscale particles using techniques such as chemical vapor deposition, electrospinning and sol–gel processing (Fig. 3). There are pros and cons to each method and they also rely on the nature of the bionanofertilizers.^42^ Electrospinning is inexpensive and easy, whereas physical methods minimize nutrient particle size through mechanical action, such as grinding or milling. Chemical methods synthesize nanoparticles through reactions such as precipitation or sol–gel, while biological methods utilize plants and microorganisms to synthesize nanoparticles.^43^

### 5.1 Top–down approach

The top–down method entails reducing bulk materials into nanostructures or smaller particles *via* physical processes, such as grinding zeolites or different minerals with significant cation exchange capacities.^44^

#### 5.1.1 Electrospinning

It uses electrical forces to draw charged polymer solutions into ultrafine fibres, offering a cost-effective and straightforward process for creating nanoparticles.^45^

#### 5.1.2 Chemical treatment

It involves using reactive chemicals to etch or reduce particle size.^46^

#### 5.1.3 Thermal extirpation

It applies high temperatures to fragment materials.

#### 5.1.4 Sputtering

It bombards a target with energetic particles to release nanoparticles.^47^

### 5.2 Bottom–up approach

The bottom–up approach entails using chemical processes, resulting in nanoparticles of decreasing size. The process involves extrapolating the procedures used to produce micro-sized particles.

#### 5.2.1 Electrochemical synthesis

It uses redox reactions at electrodes to form nanoparticles.^48^

#### 5.2.2 Precipitation

It involves mixing solutions to form insoluble nanoparticle products.^49^

#### 5.2.3 Vapor deposition

This includes chemical vapor deposition (CVD) and its variants, which operate by converting precursors into the gaseous phase and depositing them onto substrates, resulting in engineered films or coatings.^50^

#### 5.2.4 Sol–gel process

It is a wet-chemical method that offers excellent tunability, allowing precise control over nanoparticle size, shape and composition by adjusting reaction conditions such as precursor concentration, pH and temperature. It forms nanoparticles through hydrolysis and condensation of metal alkoxides. This method is especially valued for producing homogeneous, high-purity nanoparticles with tailored properties.^51^

#### 5.2.5 Molecular condensation

It assembles nanoparticles from gaseous molecules under controlled conditions (Table 2).

**Table 2:** Key synthesis methods of bionanofertilizers with examples

Method	Approach type	Bionanofertilizer/nanoparticle example	Advantages	Citation
Ball milling	Top–down physical	ZnO or TiO_2_ NFs produced from bulk oxides; co, Cr, W, Ag, and Fe nanopowder used as micronutrient sources	Simple equipment, scalable, dry process, and mixes nutrients with carriers effectively	52
Sputtering	Top–down physical	Nano-coated urea or KCl granules with thin Zn, Cu or other micronutrient films	Very uniform thin films, precise thickness control, and strong adhesion	53
Precipitation	Bottom–up chemical	Nanoscale CaCO_3_, Zn(OH)_2_/ZnO or phosphate NPs for slow-release P and Zn fertilizers	Simple wet chemistry, cheap salts, tunable size *via* pH and supersaturation	54
Vapor deposition (PVD/CVD)	Bottom–up physical/chemical	Nano-coated NPK granules *via* physical vapor deposition of micronutrient metals; nano-SiO_2_ films	Excellent control over film thickness, composition and morphology	53
Molecular (inert gas) condensation	Bottom–up physical	Nanocrystalline Fe, Cu or alloy powders that can be blended into micronutrient formulations	Produces highly pure, very fine or amorphous nanoparticles with good control	53
Sol–gel process	Bottom–up chemical	Metal-oxide NFs, such as nano-ZnO, nano-TiO_2_ and nano-silica, used as micronutrient carriers	Excellent control of composition and homogeneity; can encapsulate nutrients in porous matrices	54
Spray pyrolysis	Bottom–up chemical	Nano-layered metal oxides (*e.g.*, ZnO and Fe_2_O_3_) deposited on fertilizer granules; nano-structured K or P carriers	One-step continuous process, good control over particle size and morphology, and easily scalable	52
Laser pyrolysis/laser ablation	Bottom–up physical	High purity metal-oxide NPs (*e.g.*, SiO_2_, TiO_2_, and Fe_3_O_4_) that can be formulated as foliar NFs	High purity, narrow size distribution, and precise control through laser parameters	55
Green synthesis (plants and microbes)	Bottom–up biological	Zn nanoflowers from bacteria for agricultural use; Si NPs from bamboo; Ag and CuO NPs from plant extracts, used as nano-micronutrients or bio-stimulant fertilizers	Low-cost, aqueous, non-toxic, uses renewable biomass, and adds bioactive capping agents that may enhance plant response	56
Electrochemical processes	Bottom–up electrochemical	Nanosized metal or metal-oxide fertilizers (*e.g.*, nano-Fe or nano-Cu) generated at electrodes and collected as suspensions or coatings	Fine control of particle size *via* voltage and current; relatively low temperature; can be continuous	57

## 6 Classification of nanofertilizers

Nanofertilizers are categorized into five types: action-based, targeted delivery, plant growth-stimulating, controlled release of water and nutrients, and enhanced plant growth with reduced nutrient loading.

### 6.1 Action-based

#### 6.1.1 Chitosan-based nanofertilizers

Chitosan is a deacetylated derivative of chitin found in arthropod shells and remains highly valued in various agriculture and food sectors. It interacts with negatively charged particles and forms complexes with fertilizer molecules, thereby increasing their availability for plants. Chitosan nanoparticles enable the controlled release of NPK fertilizer in different agricultural practices and promote the uptake of growth regulators. Chitosan also functions as a carrier that protects biomolecules from extreme pH fluctuations and harsh temperature conditions.^58^ Potassium-incorporated chitosan nanoparticles reduce soil nutrient losses and help prevent land degradation. The slow-release nature of potassium from these nanoparticles enhances biomass growth in *Zea mays*.^59^ Furthermore, chitosan nanoparticles provide a sustained nutrient release that reduces fertilizer requirements and improves productivity.^60^

#### 6.1.2 Nanocapsule-based nanofertilizers

Nanocapsules are fabricated organically or inorganically and are designed for nutrient delivery. They are engineered with biopolymers and lipids alongside metal oxides in varying, complex combinations. Nutrients are released gradually from these capsules, thereby improving fertilizer efficacy and minimizing the risk of loss. Nanocapsule application boosts nutrient absorption and crop productivity, especially under stress conditions, such as enhanced maize growth with better nitrogen release profiles.^61^

#### 6.1.3 Nanogel-based fertilizers

Nanogels are sponge-like materials comprising a polymer and a liquid that absorb fertilizers and other substances, slowly releasing them subsequently. They harbor vital nutrients such as nitrogen and phosphorus, which are crucial for vigorous plant development alongside potassium. These materials serve various purposes in fertilizers and drug delivery and also help retain pure water. For the bionanofertilizer system, nanogels are essential because they combine adsorption, filtration and controlled-release processes to preserve water purity. Their nanostructured architecture allows for effective pollutant removal while promoting sustainable farming practices. Nanogels improve soil water retention and reduce erosion while being thoroughly biodegradable and essentially non-toxic. Using a specific nanogel fertilizer boosted the germination rate and improved enzyme activity and resulted in heftier fruit weight in *Abelmoschus esculentus* (okra).^62^

#### 6.1.4 Polyurethane-based fertilizers

Polyurethane-based nanofertilizers represent a novel fertilizer type crafted from polyurethane nanoparticles synthesized *via* the ring-opening metathesis polymerization (ROMP) process, which is more complex. Bionanofertilizers deliver essential nutrients like nitrogen and phosphorus, enhancing the efficiency of traditional fertilizers to a rather substantial degree.^63^ The polyurethane matrix shields particles and facilitates controlled release of nutrients, thereby enhancing nutrient efficiency and reducing erosion.

Traditional fertilizers are sometimes sheathed in polymers like polyurethane, creating slow-release options that gradually release over time in certain agricultural contexts. Biodegradable materials are often used instead of incorporating certain nutrients like sulfur due to their cost-effectiveness and properties.^64^ Turning natural polymers into biopolymers and blending them with various other substances yields a relatively affordable bio-based polyurethane rather quickly. Polyurethane-based nanofertilizers are expected to rapidly gain popularity in the global agriculture sector.^65^

#### 6.1.5 Starch-based nanofertilizers

Nanofertilizers derived from starch are crafted from the nanocrystals that readily dissolve in water and can be administered to plants *via* liquid suspensions or aerosols. Crops are fertilized without producing chemical waste.^66^ Plants treated with nanofertilizers grew taller and reactive oxygen species were reduced, with much lower metal release than those treated with alternative methods.

### 6.2 Mineral-based nanofertilizers

#### 6.2.1 Clay-based nanofertilizers

Clay nanoparticles exhibit huge surface areas and nanolayer reactivity. This makes them crucial for controlled-release fertilizer formulations, active surfaces and advantageous properties such as high specific surface area and hydrogel capacity.^67^

#### 6.2.2 Layer double hydroxides

Layer double hydroxides (LDHs) are two-dimensional compounds encompassing anionic materials that facilitate the release of anions while balancing their overall positive charge rather slowly. Varied properties, such as anion exchange and thermal stability, arise from different composition methods. It was investigated that Mg-AI LDH boosts phosphorus levels for fertilization purposes and intercalating herbicides with Mg/AI LDH enables rather slow-release formulations.^68^

#### 6.2.3 Zeolite-based nanofertilizers

Zeolites are used as soil amendments owing largely to absorption and retention of water, nutrients and various organic compounds. Zeolite-based nanoparticles exhibit efficiency and can be tailored rather specifically for crop needs, reducing fertilization costs substantially today.^69^

#### 6.2.4 Nutrient-loaded nanofertilizers

Nano-porous zeolite acts as a nutrient-rich nanofertilizer, providing crops with essential micronutrients gradually over a long period underground. Mixing volcanic ash with alkaline lake water creates a honeycombed structure that retains moisture and vital nutrients. Natural zeolite is ground into fine powder and combined with nitrogen, phosphorus, and potassium, which improves nutrient absorption.^69^ Engineered nano-porous zeolites, known as aluminosilicates, feature diverse pore sizes that enhance their effectiveness as fertilizer carriers. They increase soil fertility, help reduce erosion at soil surfaces, and decrease the need for extensive irrigation.

### 6.3 Carbon-based nanofertilizers

Carbon plays a vital role in life forms. It occurs ubiquitously in organic compounds and participates in numerous biochemical reactions. Nano biochar residual plant materials perform significant, often captivating, functions in various plants underground. Nano biochar consists of various carbon nanostructures that oxidize quickly when exposed to air, resulting in the formation of pores on its surface due to pyrolysis. Pores facilitate micronutrient uptake and store water deeply, retaining soil moisture sufficient for germination and plant development during droughts. Carbon-based nanofertilizers like carbon nanotubes (CNTs) and graphene enhance nutrient delivery, promoting overall plant growth and boosting crop yields.^70^

#### 6.3.1 Plant growth-stimulating nanofertilizers

Carbon nanotubes (CNTs) are a type of nanofertilizer that promotes plant growth by interacting with root systems and stimulating hormone synthesis. They can increase carbon and various nutrients in the soil while enhancing plant growth. CNTs absorb nutrients and release them gradually. This improves soil structure and increases water retention, which promotes plant growth. Low concentrations can increase seed germination and root development significantly without inducing phytotoxicity.^71^ CNTs penetrate deeply into the soil, providing long-term nourishment for plants while reducing the fertilizer input. Their tensile strength makes them suitable for use as a protective coating on seeds, thereby preventing pests and boosting the absorption of nutrients.

### 6.4 Functional and smart delivery systems

#### 6.4.1 Controlled-release nanofertilizers

Controlled-release nanofertilizers provide an alternative to nutrient leaching and the inefficient usage of conventional fertilizers. These nanodevices regulate nutrient release, improving absorption and reducing environmental impact. They encapsulate nutrients in a nanoscale carrier that is affected by environmental factors such as temperature, pH, and moisture and stimuli-responsive mechanisms like biodegradation and enzyme-mediated degradation.^72^ These are eco-friendly crop management solutions that improve nutrient use efficiency, reduce environmental effects and increase crop yields. They facilitate focused, extended delivery, minimizing fertilizer rates and nutrient uptake. These fertilizers can be used for most crops, such as rice, wheat, corn, and soybeans, and further studies are needed to realize their full potential in meeting global food production needs.^73^

#### 6.4.2 Nanoaptamers

Nanoaptamers serve as novel delivery systems targeting specific soil molecules and delivering essential nutrients directly into plants. Nanoparticles bind plant hormones and enzymes, thereby increasing nutrient uptake from soil.^74^ Gold nanoparticles or liposomes are attached to fertilizer components, allowing targeted delivery into plant cells. Nanoaptamers modulate the dissemination of fertilizers through root systems and soil microbes, enabling precise dosing.^75^

#### 6.4.3 Nanobead fertilizers

Nanobeads are designed to release nutrients slowly, reducing plant water loss and improving fertilization by reaching small soil cracks. Popular nanobead-based fertilizers like NanoFert and N-Flex provide essential macro- and micronutrients while minimizing harm to beneficial soil microbes and preventing over-fertilization.^76^

#### 6.4.4 Nanoemulsion-based fertilizers

Nanoemulsions are nanodroplets encapsulating a mixture of water-soluble and insoluble nutrients, mostly used in nanoemulsion-based fertilizers today. Fertilizers formulated with surfactants and high-energy mixing create stable mixtures with tiny droplets, usually smaller than 100 nm, enhancing nutrient uptake in plants. Adding 1% paraffin oil nanoemulsion boosted biomass yield and biochemical content in the microalgal strain *Chlorella pyrenoidosa*. Nanoemulsions can be integrated into traditional irrigation setups and help eradicate fungal pathogens.^63^

#### 6.4.5 Water and nutrient loss-controlling fertilizers

Bio-nanofertilizers can reduce water loss. Designing these bio-nanofertilizers involves porous matrix encapsulation or hydrophilic surface modification.^77^ Ammonium sulfate, potassium, zinc, copper, molybdenum, boron, iron oxide, sulfur and calcium are a few examples of nanofertilizers. Nanobeads and nanoemulsions are also utilized.

### 6.5 Nutrient-based nanofertilizers

#### 6.5.1 Organic nanofertilizers

Organic nanofertilizers utilize nanoparticles derived from organic matter, enhancing plant growth by retaining moisture and regulating pH levels for improved nutrient absorption. Eco-friendly fertilizers can be obtained from various natural sources and crafted into different forms, such as capsules and polymer conjugates.^78^ Gums and seed polysaccharides are commonly used in production, along with other exotic natural polymers. Organic bionanofertilizers improve the soil structure and promote water infiltration, aeration, and deeper root growth.^79^ Organic materials in fertilizers support soil microorganisms, which boost nutrient cycling. NanoMax-NPK and Ferbanat bionanofertilizers contain probiotics, amino acids, vitamins, and organic micronutrients, strongly enhancing root zone activity.

#### 6.5.2 Inorganic nanofertilizers

Inorganic nanofertilizers consist of metals, metalloids and non-metallic nanoparticles that provide critical nutrients such as nitrogen, phosphorus and potassium to plants. They enhance nutrient absorption efficiently and boost agricultural yields under optimal conditions. These products fall under macronutrient or micronutrient-based fertilizers.^7^

#### 6.5.3 Macronutrient-based nanofertilizers

Plants require various macronutrients like magnesium, nitrogen, sulfur, phosphorus, and calcium for growth. A deficiency in certain nutrients severely hampers growth and makes plants highly susceptible to infection and disease. A delicate balance of macronutrients promotes healthy plant growth in various environments. Fertilizers with main macronutrients like nitrogen, phosphorus and potassium are typically labelled as NPK.^13^

Nanofertilizers made of nitrogen enable plants to grow because of the slow release of nitrogen. Nitrogen plays an important role in the production of amino acids, nucleic acids and chlorophyll. Its deficiency leads to stunted growth and discoloration of leaves.^80^ Nanofertilizers are superior to the standard urea and contribute to the germination of plants. They achieve this with nanoparticles, which are modified chips of urea-coated zeolites.^81^ Photosynthesis depends on nanofertilizers that are based on magnesium. They enhance the production of crops like sesame, particularly in arid soils.^82^ Phosphorus is essential in most growth processes. It increases wheat production and accelerates the growth of the plants. Apatite nanoparticles have the capability of boosting seed production by 20% and growth rate by 30%.^83^ Potassium nanofertilizers enhance the absorption of nutrients. They assist crops such as alfalfa and daffodils to better withstand stress and contribute to long term productivity. They are not easily soluble and this assists in stabilizing nutrition.^84^ The nanofertilizers of sulfur have been known to increase nutrient transfer and the growth of the plant.^85^ Their other benefits include the enhancement of resistance to diseases and the ability to enhance the use of nitrogen.^86^ Nanofertilizers obtained with the help of calcium catalyze an increases in crop yields and disease resistance considerably. They have increased the biomass in peanuts by 15%. The types of nanofertilizers are very important in the modern farming industry. They promote both sustainable and efficient farming.^87^

#### 6.5.4 Micronutrient nanofertilizers

Micronutrients are essential for plant growth and numerous metabolic functions occurring within cells. Zinc acts as a cofactor for various enzymes and proteins, facilitating defence responses, auxin regulation, carbohydrate synthesis, and protein metabolism. Boron promotes cell wall biosynthesis and overall plant development through multiple pathways, such as lignification in many plant species.^88^ Research indicates that nanofertilizers containing Zn or B increase fruit yields in sunflower and sugarcane, particularly under different agricultural conditions.^30^ Manganese supports enzyme activity and photosynthesis, whereas iron is essential for plant growth. Studies on nanoscale materials such as zinc, copper, manganese and iron oxide reveal enhanced germination and plant growth at relatively low dosages. These impact productivity and require proper supplementation for optimal yields.^88^

Iron nanofertilizers contain iron, and they enhance the amount of iron in soils with low natural solubility. They consist of nanosized iron, non-encapsulated forms, and nanocomposites, and they offer considerable benefits to crop production and reprocessing iron-deficient soils.^89^ Nanofertilizers that are made using copper are also effective in delivering nutrients to plant cells, which enhance growth and enhance the yields of maize and wheat.^90^ They are safe for human beings and animals, have antibacterial effects that aid plants against pests and can be afforded by small-scale farmers, but highly concentrated ones possess phytotoxicity and need to be handled with care.^91^ Boron is vital to cell-wall development and movement of carbohydrates. Nanofertilizer additions of boron boost biomass in lettuce and zucchini and enhance the volume of pomegranates, commonly together with zinc nanofertilizers.^92^ They may be used as foliar sprays in the supply of micronutrients.^93^ Manganese is a cofactor in several stress responses, and it is crucial to the formation of secondary metabolites.^94^ Compared with traditional MnSO_4_, a concentration of 0.05 mg L^−1^ was demonstrated to deliver nutrients to seedlings and enhance the growth and photosynthesis of mung-bean seedlings.^95^

#### 6.5.5 Hybrid nanofertilizers

Hybrid nanofertilizers enable the slow release of nutrients, thereby improving access by combining traditional fertilizers with nanotechnology-based fertilizers.^96^ Nanocomposites are obtained by blending a polymeric phase with a dispersed nanofiller, resulting in superior performance due to enhanced mechanical properties. Hybrid nanofertilizers can significantly reduce fertilizer use in plants, leading to substantial cost savings for farmers today.^97^ Nano-fertigation involves covertly introducing nanoparticles as fertilizers into irrigation systems, thereby increasing nutrient absorption while minimizing waste. A variety of hybrid nanofertilizers are emerging, including nano-encapsulated products and more advanced bionanofertilizers with specific benefits (Table 3).

**Table 3:** Comparative features of bionanofertilizers

Material class	Composition/Structure	Mechanism & kinetics	Agronomic advantages	Risks/limitations
Carbon-based (biochar, CNTs, and graphene)	Biochar with nano-porosity; carbon nanotubes (CNTs) or graphene acting as nano-carriers for NPK/micronutrients	Sorption-desorption from porous surfaces; relatively slow, moisture and charge-dependent release	Improved water holding and root colonization; enhanced photosynthesis and germination	Synthesis cost for engineered carbons; potential for high concentrations to impact soil biota if not optimized
Chitosan-based	Nano-chitosan or chitosan-NPK composites; cationic biopolymer matrix	Diffusion through biodegradable polymers; pH and enzyme-responsive slow release	Protection of biomolecules from pH/temp fluctuations; improved biomass in *Zea mays* and stress mitigation	Sensitivity to environmental pH; potential cost and scale-up challenges
Clay/layered silicate	Nano-clays with high surface area and hydrogel capacity	Ion exchange and interlayer diffusion are relatively slow and moisture dependent	Strong nutrient retention; improved soil structure, water holding and aeration	Potential swelling/dispersion issues; high energy demand for physical size reduction (grinding)
Layered double hydroxides (LDHs)	Mg–Al LDH 2D structures with intercalated anions (*e.g.*, phosphate or herbicides)	Anion exchange-controlled release; slow to moderate, depending on soil chemistry	Enhanced phosphorus availability; tunable release; dual delivery of nutrients and herbicides	Synthesis complexity; persistence in soil depends on layer composition
Nanocapsules/polymer coatings	Organic shells (*e.g.*, polyurethane, starch, and biopolymers) encapsulating nutrients	Diffusion through or degradation of the shell; designed release (40–90 days possible)	High nutrient use efficiency; better synchronization with crop demand; reduced application labor	Potential microplastic concerns for non-biodegradable shells, like certain polyurethanes
Zeolite/nanoporous carriers	Natural or engineered aluminosilicates loaded with NPK	Ion exchange and diffusion from pores; long-term, very slow release	Massive reduction in nitrogen leaching; improved soil fertility and water retention	Physical mining footprint; limited effectiveness in highly specific soil chemistries
Macronutrient nan-formulations	Nano-urea, nano-apatite (P) and nano-K systems	Matrix-controlled or coating-mediated; gradual release compared to salts	Higher NPK use efficiency (up to 70% for N); improved chlorophyll and biomass	Risk of phytotoxicity if over-dosed; requires crop-specific dose optimization
Micronutrient nano-formulations	Metal/oxide nanoparticles (ZnO, Fe, Cu, B, and Mn)	Fast to moderate release; often *via* foliar spray or seed-priming with rapid uptake	Correction of micro-deficiencies; yield gains, improved stress tolerance and enzyme activation	Narrow margin between beneficial and toxic doses; potential for bioaccumulation
Hybrid/loaded systems	Urea-HAP hybrids; nano-zeolites N; nano-fertigation mixtures	Composite release: Initial pulse followed by slower nano-mediated release	Substantial fertilizer saving (20–50%) compatible with existing irrigation	Formulation complexity; potential infrastructure requirements for nano-fertigation

## 7 Mechanism of bionanoparticle uptake in plants

**Fig. 4:**
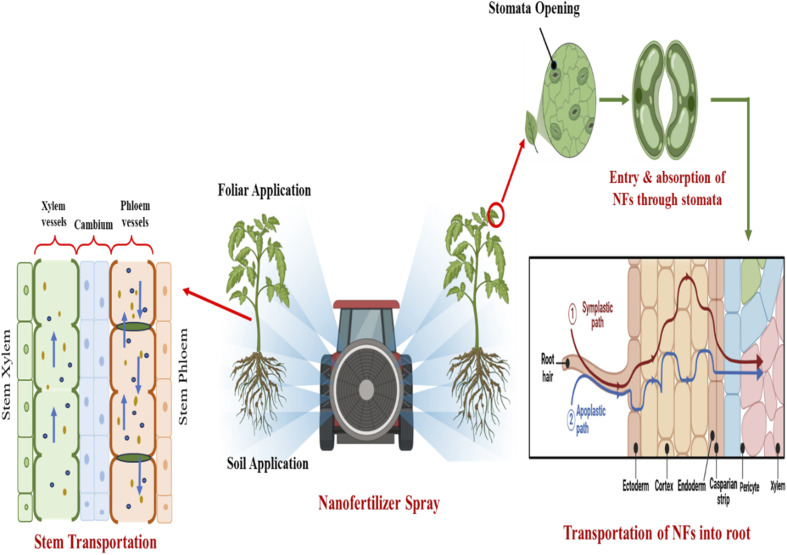
Mechanism of bio-nanofertilizer uptake in plants (BioRender (https://www.biorender.com/).

Bionanoparticle uptake and distribution in plants are strongly influenced by factors such as plant species, growth conditions, and nanoparticle physicochemical properties. Bionanofertilizers enter plant tissues through roots or upper parts, with size, shape, and interaction with cell walls critically influencing absorption processes.^98^ Surface-functionalized nanoparticles can significantly boost the uptake of larger particles by altering pore size, but the cell wall size exclusion limit remains a substantial barrier. Nanocarriers protect nutrients and extend their presence in soil, while foliar application allows for deep penetration through plant stomata or fuzzy trichomes. Nanoparticles within plant cells are transported between cells *via* vascular systems or through plasmodesmata more quickly (Fig. 4).^99^

Nanoparticles have a substantial regulatory impact on plant growth at moderate levels, yet can induce negative effects at rather high concentrations. The unique physicochemical properties of nanoparticles largely dictate their environmental fate and subsequently influence overall ecotoxicity in various ecosystems. Various nanoparticles induce phytotoxicity or genotoxicity depending heavily on the structure and specific surface functionalization. The phytotoxicity effects of nanoparticles are affected by their availability in soil interactions.^100^

## 8 Interactions of nanofertilizers with plant cells

Nanoparticles exhibit unique biological, chemical and physical properties due largely to their high surface area-to-volume ratio. Imaging techniques like transmission electron microscopy and confocal microscopy are used to observe interactions, not to facilitate them. Confocal microscopy enables three-dimensional (3D) imaging of plant cells and tissues, allowing researchers to visualize structures deep within thick samples without physical sectioning. This technique is potent for studying living cells, dynamic processes and spatial relationships within intact tissues, such as cytoskeletal organization, calcium signaling and symplastic connections. It uses fluorescence to highlight specific cellular components, providing clear images with high depth discrimination by eliminating out-of-focus light. It facilitates 3D reconstructions, which are essential for understanding the spatial arrangement and interactions of organelles and biomolecules in large or complex plant cells.^101^ Transmission electron microscopy (TEM) offers ultrastructural detail at nanometer resolution, revealing the fine architecture of cell walls, membranes, organelles and subcellular compartments. This technique is essential for characterizing the ultrastructure and compositional heterogeneity of plant cell walls, including the identification of distinct cell layers and the localization of specific components such as lignin and cellulose. It provides high-contrast images of thin sections, enabling the study of structural changes at the molecular level that are not visible with light-based techniques.^102^ Plants absorb relatively larger nanoparticles *via* stomata and smaller ones through various lipophilic cuticular or oddly hydrophilic alternative pathways.^103^

The phloem system serves as the primary pathway for nanoparticle transmission from leaf surfaces downwards, involving various peculiar transport mechanisms like aquaporins and membrane transporters. Polymer-coated bionanofertilizers, such as alginate and polyacrylates, act smartly, releasing nutrients slowly in a very controlled manner.^104^ Chitosan-coated bionanofertilizers and nanoclay-based fertilizers show increased effectiveness in releasing nutrients, but rather slowly compared with conventional fertilizers. Urea-hydroxyapatite hybrid bionanofertilizers and natural zeolites build nutrients in the soil and release them gradually over time.^105^

Nanofertilizers ooze into plants *via* diverse routes, including apoplastic pathways, symplastic routes and transmembrane channels rapidly underground. Particle size varies greatly from 1 nm to 100 nm and they generally exhibit a more positive surface charge overall. Bionanofertilizers accumulate variably in crop plants due largely to differing particle characteristics and drastically altered rhizosphere composition around various plant types. Application methods affect how well nanofertilizers work on different plant types. The application method influences the effectiveness of bionanofertilizers on plant growth under various conditions.^27^

## 9 Mode of bionanofertilizer application

Nanofertilizer application methods suit regions with high nutrient retention capacities under relatively consistent precipitation patterns. Soil quality and nutrient availability heavily influence method selection, and harsh climate conditions can further hamper nutrient uptake. Improving crop yield significantly depends on understanding certain underlying factors, which in turn promote sustainability and reduce environmental degradation radically (Table 4).^106^ There are three primary methods currently applied for bionanofertilizers.

**Table 4:** Types of bionanofertilizers, their application, assessments on crop growth and environmental impact

Bio-nanofertilizer			Impact assessment		Citations
Type of bio-nanomaterial	Nutrient delivered	Application method	Impact on growth	Environmental effect	
Carbon-based nanoparticles	Nitrogen	Foliar spray	Improved chlorophyll content and yield	Positive	22
Metal oxides (ZnO)	Zinc	Seed coating	Enhanced root growth and nutrient uptake	Positive	12
Iron nanoparticles	Iron	Soil application	Increased plant biomass and yield	Positive	22
Titanium dioxide (TiO_2_)	None (enhances photosynthesis)	Foliar spray	Boosted photosynthesis	Neutral	107
Polymeric nanocarriers	Phosphorus	Controlled release in soil	Reduced nutrient loss and improved growth	Positive	108

### 9.1 Foliar spray method

The method of foliar application involves directly spraying bionanofertilizers onto plant leaves for rapid nutrient absorption, which is especially advantageous in low-fertility areas. This method applies liquid fertilizers to leaves or foliage, enabling rapid nutrient uptake through the leaf surface. It utilizes targeted delivery of bionanofertilizers to ensure precise and effective transfer of essential elements such as bionanofertilizers, fungicides, herbicides, and preservatives. The absorption process depends on the size of particles relative to leaf wax and cell walls, with most nanoparticles accumulating in vacuoles.^109^ Plant traits, nanoparticle physical properties, and environmental conditions influence absorption and transport. Foliar sprays offer several benefits, including faster response, higher nutrient utilization, and reduced leaching and runoff. Studies have shown that foliar application of bionanofertilizers can enhance nutrient uptake, stimulate plant growth, and increase crop yields. For example, cerium oxide (CeO) and carbon-based nanoparticles improved wheat production by 36.6%, bitter melon yield by 28%, and tomato fruit yield by 80%. However, its effectiveness can be affected by environmental factors such as temperature and humidity.^27^

### 9.2 Seed nanopriming

**Fig. 5:**
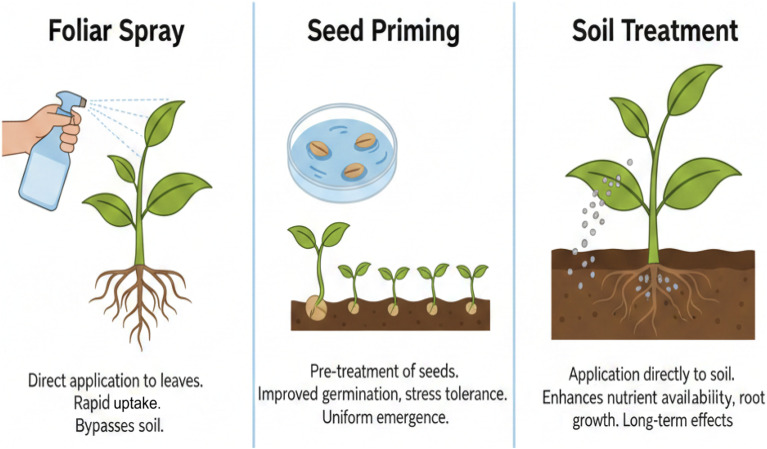
Diverse application methods of bionanofertilizers for enhanced crop productivity (https://www.biorender.com/).

Seed priming is a pre-sowing treatment that brings about physiological changes in seeds, leading to faster germination and plant growth by regulating metabolic and signaling pathways. Nanobiofertilizers promote growth by entering seed pores, spreading within the seed, and triggering plant hormones. The absorption of nano-compounds at the cellular level reduces input requirements and speeds up germination, even in poor soils. As a result, stronger seedlings emerge with better nutrient absorption. However, it is crucial to determine the optimal concentrations of nanofertilizers carefully, as excessive use can lead to phytotoxicity under different environmental conditions. Modern seed nano-priming techniques involve applying bionanofertilizers directly to the seed surface, which in turn hinders pathogen penetration (Fig. 5).^110^

Seed priming improves gene expression, particularly concerning plant resilience, leading to increased resistance. Bean seed priming with chitosan nanoparticles enhances germination and radicle length, whereas proline, chlorophyll a, and activities of antioxidant enzymes increase under salt stress. Plant stress is moderately mitigated by bionanofertilizers, which regulate hormone functions and boost antioxidants while significantly reducing Reactive Oxygen Species (ROS) production.^111^

### 9.3 Soil treatment

Soil application remains the primary method for nutrient supplementation in plants. Key considerations include fertilizer longevity, soil texture, salinity, plant salt sensitivity, soil composition, and pH. Mixing bionanofertilizers into the soil increases nutrient availability.^112^ Soil treatment involves integrating bionanofertilizers into the soil through methods such as broadcasting or localized placement, promoting slow nutrient release and minimizing losses.^113^ This method is appropriate for regions with effective nutrient retention and steady rainfall; however, it demands careful management to avoid environmental pollution and ecological imbalances. Bionanofertilizers can be applied using conventional methods like broadcasting or side-dressing, which can significantly alter their overall behavior (Table 5). Nutrients are released gradually from bionanofertilizers, substantially enhancing plant growth, and crop productivity increases markedly under such conditions.^67^

**Table 5:** Comparative evaluation of the potential benefits and drawbacks of the traditional method of agricultural production by nanotechnology

Types of agricultural production	Plant growth	Soil rhizosphere	Citations
Nanofertilizer-mediated agriculture	•High production	•More microbial diversity	114
	•Saves natural resources	•Reduces demand for fertilizers	
	•Sustainable	•Enhances native nutrient mobilization	
	•More nutritious food	•Enhances the soil's biological health	
Bulk fertilizer mediated agriculture	•High production rate	•Low microbial diversity	115
	•Affects natural resources	•Diminishes soil biological health	
	•Bioaccumulation of harmful fertilizer byproducts	•Eutrophication	
	•Decreased nutritional quality		
Organic agriculture	•Nutritious food	•Improves soil biological health	116
	•Biofertilizers can cause pathogenic signs		
	•Low production rate for demand		
Conventional agriculture	•Nutritional deficiency	•Low native nutrition mobilization	117
	•Low productivity	•Low microbial diversity	
	•Stress and disease sensitive	•Poor soil biological health	

## 10 Potential development of bionanofertilizers

Slow-release fertilizers don't reduce microbial populations. They're designed to provide nutrients gradually without harming soil microbes. They provide a nutrient supply over a specific period, offering higher uptake efficiency, minimizing capital and labor outlay, providing less frequent application, improving handling, reducing environmental pollution, and allowing flexibility in release periods of 40–90 days. Controlled-release fertilizers aren't always higher solubility compounds. The release mechanism is more about the coating, not solubility.^118^

Encapsulation of nutrients with nanomaterials can be achieved in three ways:

• Encapsulating nutrients with different chemical compositions.

• Applying nanomaterials as a thin layer coating on nutrient particles, or delivering nutrients as emulsions.

• Other available nanofertilizer designs include quick-release, specific-release, ultrasound release, heat release and pH release.

• These methods release nutrients through various mechanisms, such as contact with surfaces, chemicals, enzymes, ultrasound frequencies, magnetic fields, water, heat and acid or alkaline conditions.^117^

## 11 Impact of bionanofertilizers on yield and production factors

Crops have reaped benefits from yield-related traits courtesy of the bionanofertilizer application. Studies reveal that nano-micronutrients outperform chemical fertilizers in amplifying yields markedly. Replacing traditional fertilizers with nanofertilizers has resulted in considerably improved yields in a variety of crops, including rice and wheat.^119^ Research suggests that bionanofertilizers can boost pulse yields sustainably under certain conditions. Introducing nano-sized zinc and iron ions increases the height and branch count in chickpeas, for instance. Nano magnesium oxide improved shoot-root length, fresh biomass and chlorophyll content in horse grams.^120^ Research revealed that a mix of silver and zinc nanoparticles boosted seed yield in green gram crops. Barley plants exhibited markedly improved photosynthesis under conditions where pigment production was sped up and RuBisCO activity was enhanced. Bionanofertilizers can enhance nitrogen metabolism processes rapidly and bolster the synthesis of certain growth regulators alongside cell proliferation. TiO_2_ nanoparticles accelerate germination speed, whereas excessive ZnO nanoparticles promote stronger roots that absorb large amounts of water and nutrients.^121^

## 12 Abiotic and biotic stress tolerance of nanofertilizers

Abiotic and biotic stress reduce crop yields, adversely affecting plant development and threatening global food security. Abiotic stresses include drought, flooding, extreme heat, hail, high salinity, heavy metal toxicity and lack of essential minerals. Biotic stresses include insect pests and diseases. Crop production must surge by 70% by 2050, according to rather ambitious plans laid out by the Food and Agriculture Organization. Researchers explore novel technologies like nanoparticles to increase plant antioxidant compounds and mitigate harsh environmental stress effects nowadays.^122^

### 12.1 Abiotic stresses

Water scarcity intensifies worldwide, hampering agricultural output drastically under severe drought conditions. Cultivating drought-resilient crops remains vital, but deploying stress-ameliorative materials such as bioengineered nanoparticles holds tremendous potential amidst severe water scarcity. Nanoparticles can strengthen soil water retention and mitigate the production of reactive oxygen species (ROS) that lead to cellular damage or death. Nanoparticles substantially boost antioxidant levels and proline, thereby reducing the formation of harmful compounds like H_2_O_2_ in bodily fluids. Silicon nanoparticles bolster drought tolerance in hawthorn seedlings largely by underpinning various physiological and chemical functions vital for their survival.^119^ Zinc oxide nanoparticles (ZnO nanoparticles) noticeably boosted soybean germination rates under severe water stress, while iron nanoparticles mitigated their harsh effects on safflower yield and oil content.^123^ Titanium dioxide (TiO_2_) nanoparticles enhanced wheat crop performance under drought conditions, boosting tiller numbers substantially and grain yield concurrently. Silver nanoparticles markedly enhanced lentil germination and root growth significantly under severe drought stress. Combining iron nanoparticles with salicylic acid increased the strawberry drought tolerance and wheat yields were enhanced by using nano-chelated nitrogen fertilizers. Nano-zeolite-loaded nitrogen application reduced the sage growth inhibition caused by water stress more under various experimental conditions.^122^

High concentrations of Na^+^, Cl^−^, and SO_4_^2−^ ions in soil severely reduce osmotic potential and hinder plant growth, ultimately causing death. Arid regions face this challenge with significant severity, affecting cultivated lands worldwide at an alarming rate of over 20%. Salt stress greatly disrupts various physiological processes, including photosynthesis and nutrient uptake balance in plants, leading to rapid ionic toxicity.^123^ Nanofertilizers have recently surfaced as a potential advanced solution in global agriculture. Research indicates that applying SiO_2_ nanoparticles increases leaf dry weight and vital contents like chlorophyll and antioxidants under salinity stress, thereby alleviating Na^+^ toxicity and supporting plant growth. Maize grown under salt stress shows higher yields with SiO_2_ nanoparticles, while nano-calcium enhances fruit yield in *Solanum lycopersicum* compared to traditional fertilizers.^124^ Foliar application of nano-NPK has produced more promising results for pea plants under salinity stress. These advancements highlight nanotechnology's potential to strengthen plant resilience against salinity.^124^

Extreme temperatures cause severe oxidative stress in plants, negatively affecting photosynthesis and reducing chlorophyll levels, which stunts growth. Heat stress leads to the excessive production of reactive oxygen species, damaging membranes more severely and causing ions to leak rapidly. Low doses of selenium nanoparticles somewhat lessen heat stress effects by improving water relations and boosting antioxidant activities. Plants produce heat shock proteins when temperatures increase, and multiwalled carbon nanotubes enhance gene expression for these proteins.^125^ Wheat treated with silver nanoparticles showed notable growth improvements under heat stress, including increased root length and shoot weight. Applying Se nanoparticles to sorghum under high-temperature conditions increased pollen germination and seed yield during trials. Abiotic stress triggers an increase in antioxidant levels and crop yield quality, with nanoparticles reducing the formation of some harmful compounds.^126^

### 12.2 Biotic stresses

Annual yield losses from diseases and pest infections worldwide are estimated at roughly 20%, or maybe even as high as 40%. Farmers yearly deploy vast amounts of pesticides, leading to environmental pollution, food toxicity, and a decline in soil fertility. Recent studies suggest that nanoparticles or nanofertilizers combat soil and plant pathogens by penetrating microbial cells disruptively. Nano-Cu has shown considerable efficacy against bacterial diseases in mung crops and rice caused by blight. Research documented the effectiveness of Cu–Zn bimetallic nanoparticles against yeast and chitosan nanoparticles were highlighted for controlling various diseases by binding microbial cells.^127^ Chitosan–Cu complexes and chitosan–saponin complexes exhibited potent fungicidal activity against various fungal species at a relatively low 0.1% concentration. MgO nanoparticles impeded the growth of *Ralstonia solanacearum*, while Cu-based nanoparticles exhibited considerable antimicrobial potential against various pathogens.^128^ Nanoparticles and bionanofertilizers are reducing harmful microbial growth across various agricultural fields. Nanofertilizers might utterly transform agroecosystems by massively enhancing nutrient uptake efficiency and greatly improving crop productivity. They mitigate nutrient runoff and greenhouse gas emissions, promoting sustainability in the environment while tackling agricultural challenges *via* improved soil health.^129^

## 13 Applications of bionanofertilizers

Nanoparticles hold significant promise in fields such as horticulture and pharmaceuticals, but they also present unknown risks to human health and the environment. When used alone, nanoparticles can promote plant growth by enhancing processes like photosynthesis and nitrogen fixation. They increase the effectiveness of biofertilizers, resulting in higher crop yields and improved nutrition. Neem cakes and beneficial microbes have helped *Vigna radiata* seeds grow better with nanostructured NPK fertilizers.^130^ Combining bionanofertilizers with Cu nanoparticles encourages plant growth. A notable rise in *Zea mays* grain production was observed after using bionanofertilizers, with results showing better vine growth and berry quality when used together with the *Ascophyllum nodosum* biofertilizer compared to bionanofertilizers alone.^131^ Nanoclay-coated biofertilizers improved yield attributes in *Vigna* by improving water retention and nutrient efficiency.^132^

## 14 Bionanofertilizer functions in hydroponically grown plants

Hydroponics is a method of cultivating plants and crops with minimal space requirements. Nanofertilizers are frequently employed in hydroponically produced crops, which have magnetic nanoparticles in roots, stems, and leaves. While nanoscale Zn and ZnO nanoparticles may reduce seed germination in some plants, zucchini seed germination and root development revealed no adverse effects. *Prosopis velutina* plants have elevated catalase and peroxidase enzyme activity, showing a high tolerance to nanoparticles.^133^ Higher doses of hydroxyapatite nanofertilizers did not affect germination but did promote root elongation. To enhance atonikin, hydroponically produced nano-silicon (NS) fertilizers and nano-complete (NC) fertilizers and barley fodder were utilized.^134^ The findings indicated that plants cultivated in an NS + A + NC hydroponic solution exhibited greater dry matter content, crude protein, crude fibre, acid detergent fibre and neutral detergent fibre compared to untreated plants. Cucumber plants cultivated hydroponically with N-doped amorphous calcium phosphate nanoparticles (ACP-NPs) demonstrated similar development in root and shoot biomass without nitrogen exhaustion.^135^ The viability of N-doped ACP as a nanofertilizer is backed by its excellent nitrogen utilization efficiency and economical production process. Salt stress has become a significant danger to agricultural output and food availability, owing to climate change and the swift population increase. Bionanofertilizers like nano K_2_SO_4_ have been shown to improve plant parameters, but both genotypes showed increased electrolyte leakage and relative water content.^136^

## 15 Advantages and limitations of bionanofertilizers in agriculture

Bionanofertilizers, which combine beneficial microorganisms with nanotechnology, offer several key advantages that address the challenges of feeding a rapidly increasing world population. Enhanced crop yields and productivity in bionanofertilizers improve seed germination, plant growth and crop yields by providing targeted, controlled and efficient nutrient delivery to plants.^22^ Their ability to increase nutrient use efficiency (NUE) means that crops absorb more nutrients with less input, supporting higher productivity on existing farmland. Improved soil health and sustainability fertilizers enhance soil quality by promoting beneficial microbial activity and reloading essential nutrients, leading to healthier soils over the long term. They also aid in bioremediation, helping to restore degraded soils and reduce the reliance on chemical fertilizers that can harm the soil structure and biodiversity.^137^ Reduced environmental impact by bionanofertilizers minimizes nutrient runoff and leaching, which are major sources of water pollution and ecosystem disruption associated with traditional fertilizers. Their controlled-release properties lessen the frequency and quantity of fertilizer applications, lowering the risk of soil and water contamination and greenhouse gas emissions. Increased stress tolerance and disease resistance by bionanofertilizers can boost plant tolerance to abiotic stresses (such as drought and salinity) and enhance resistance to diseases, making crops more resilient to changing climate conditions and resource limitations.^32^ They activate plant defense mechanisms and upregulate genes involved in stress responses, further supporting stable yields under adverse conditions. Cost effectiveness and resource efficiency, with higher nutrient uptake efficiency, allow for reduced application rates compared to conventional fertilizers, making them more cost-effective in the long run. Lower input requirements and fewer applications translate to reduced labor and resource use, which is particularly beneficial in regions with limited access to agricultural inputs. By contributing to food security by increasing yields, improving soil health and minimizing environmental harm, bionanofertilizers help ensure a sustainable and reliable food supply for a growing global population.^138^ Their eco-friendly nature supports both food security and safety, addressing concerns about the long-term impacts of chemical fertilizers on human and environmental health.

**Fig. 6:**
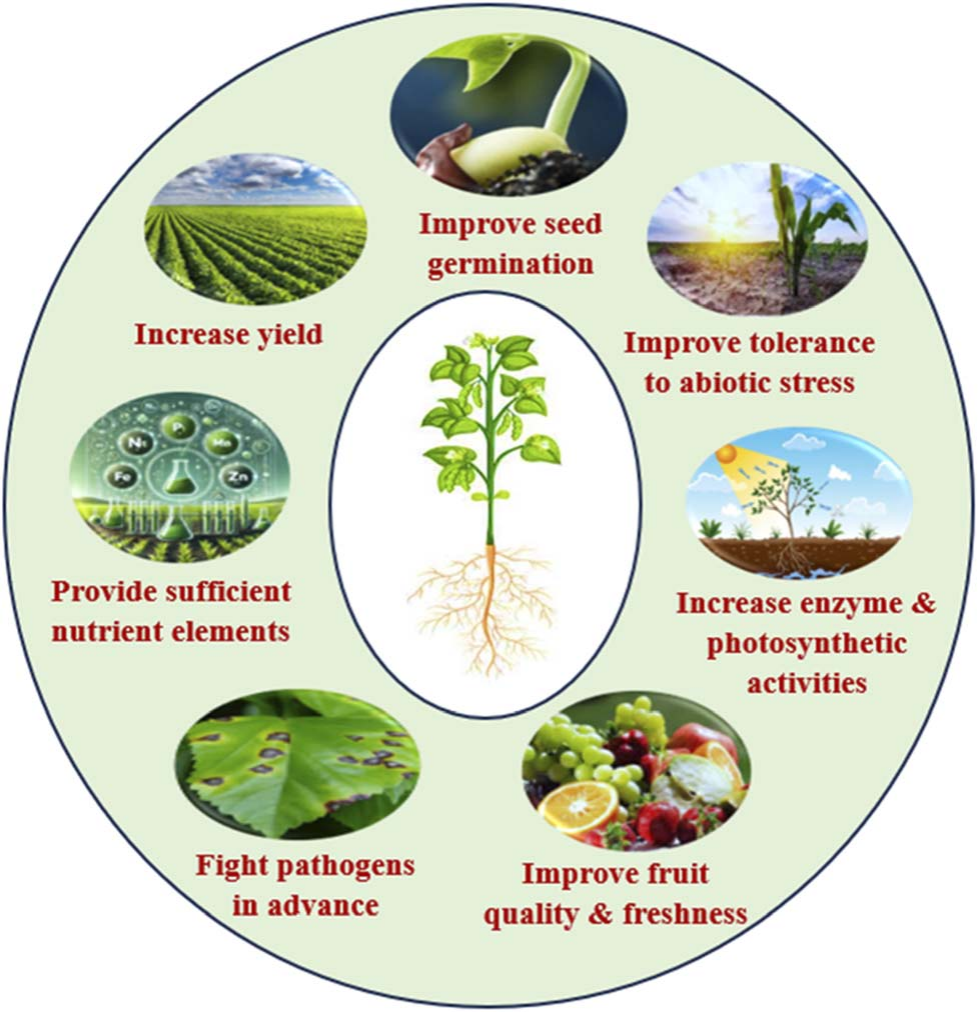
Advantages of bionanofertilizers in agriculture.

Bionanofertilizers provide several benefits in agriculture, such as lowering environmental impact, improving soil health and delivering nutrients more accurately. Nanoparticles help retain soil nutrients and reduce pollution and greenhouse gas emissions. They also boost soil microbial activity and structures, enhancing fertility and health while ensuring efficient nutrient use.^104,139^ They enhance the release of nutrients and boost absorption, thereby reducing the need for higher doses and mitigating the impact on the environment. Bionanofertilizers boost plant growth and yield exceptionally well in various crops like cereals and fruits.^140^ They provide disease resistance and pest resistance and some possess antimicrobial properties that shield plants from various pathogens (Fig. 6). Higher productivity results partly from better crop performance over time under certain conditions. Bionanofertilizers promote robust crops and reduce excessive fertilizer usage.^141^

### 15.1 Limitations and drawbacks of using bionanofertilizers

**Fig. 7:**
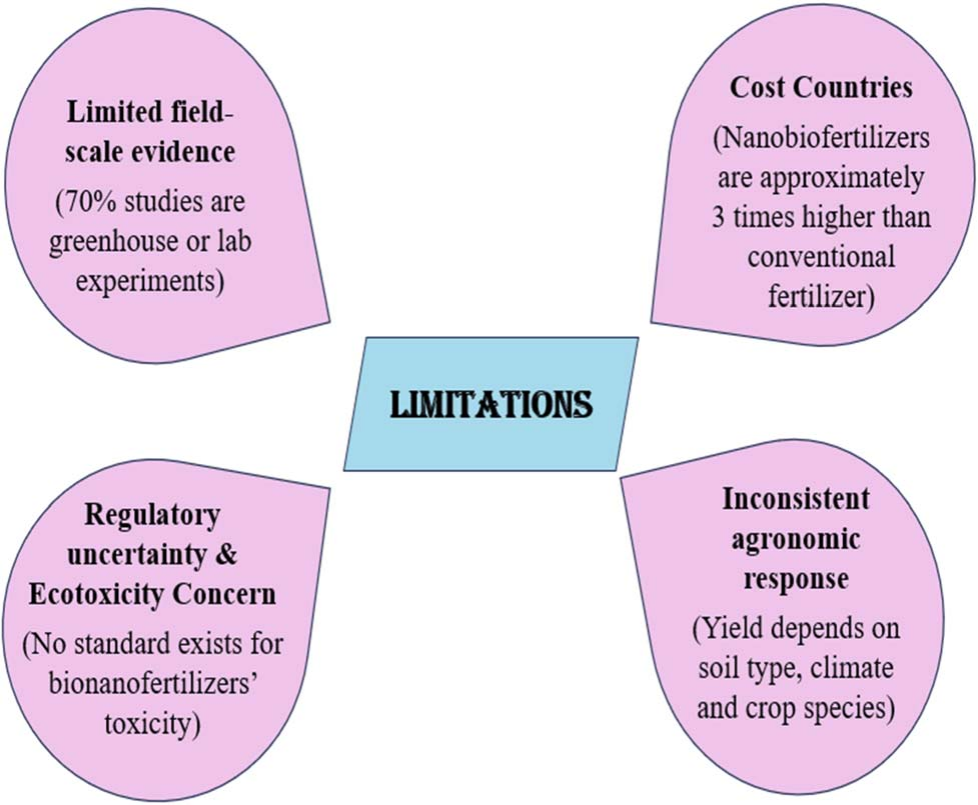
Limitations of bionanofertilizers in agriculture.

Bionanofertilizers are used as fertilizers to enhance agricultural output and improve plant nutrients. However, risks related to toxicity, along with a lack of research, regulations, and monitoring, limit their broader application. Understanding biodegradability and biomagnification transfer effects is vital for assessing toxicity. Currently, there is no suitable legislation or risk management system to monitor the use of nanofertilizers in addressing sustainable crop production challenges.^142^ Bionanofertilizers are not produced in sufficient quantities for widespread use as plant nutrients. Their higher costs and lack of standardization create challenges. Nanotoxicology assesses the harmful effects of such materials and develops safe usage practices vigorously. Comparing the safety and toxicity of various nanomaterials poses challenges due largely to factors such as size and shape and chemical composition variability. Previous studies highlighted a pressing need to link toxicological properties with specific nanoparticles at particular times accurately. Some research indicates that nanoparticles might lead to harmful biological reactions, including severe inflammation and significant DNA damage.^143^ Nanoparticles can rather bolster plant growth by heightening stress resistance and ameliorating overall health *via* the application of bionanofertilizers. New nanofertilizers must undergo rigorous validation and regulation thoroughly before marketing, ensuring safety for the environment and human health. The behavioral toxicity of nanoparticles depends on various factors like size, materials used and dosage administered irregularly. Higher concentrations of nanomaterials can harm plants, while lower doses can be beneficial under certain conditions.^144^ High dosages of engineered nanofabrics (>500 mg L^−1^) are harmful, but lower doses (50 mg L^−1^) are beneficial.^145^ High levels of ZnO nanoparticles block plant roots, causing nutrient loss. Chemical-derived nanoparticles can be toxic by creating hazardous byproducts. To solve this, there is a trend toward the biosynthesis of nanoparticles. Nanoparticles are safe for soil microorganisms but harmful to marine microflora. The FDA has stated that the safety of NP products for human use is uncertain (Fig. 7).^146^

## 16 Existing challenges for nanofertilizers

Quality control and consistency are persistent issues with the availability of poor-quality bioinoculants in the market, which undermines farmer confidence and the effectiveness of bionanofertilizers. Ensuring consistent production quality at an industrial scale remains difficult, partly due to the complexity of combining biological and nanomaterial components. Shelf life and stability in traditional biofertilizers already suffer from poor shelf life and reduced stability in the field, especially under varying environmental conditions (temperature, radiation, and pH). Bionanofertilizers, while sometimes improved by nanoencapsulation, still face challenges in retaining the viability and effectiveness of the biological component during storage and after application.^14^ Slow response and efficacy in bionanofertilizers often show a slower response compared to chemical fertilizers, which can discourage adoption by farmers seeking immediate results. Their performance can be inconsistent due to environmental sensitivities and the need for improved formulations tailored to specific crops and soils. Production and commercialization barriers in industrial-scale production are limited by technical and economic constraints, including the need for advanced manufacturing processes and regulatory approval. Timely supply and distribution of high-quality bionanofertilizer cultures can be challenging, especially in regions with less developed agricultural infrastructure. Environmental and health concerns associated with bionanofertilizers are designed to be eco-friendly. The use of certain nanoparticles (*e.g.*, carbon nanotubes and metal oxides) raises concerns about soil accumulation, potential toxicity to soil microbiota and possible entry into the food chain also posing risks to human health and the environment. The long-term ecological impact of widespread nanoparticle use in agriculture remains insufficiently studied, necessitating further research and regulatory oversight.^147^ Field performance and environmental sensitivity to the effectiveness of bionanofertilizers can be highly variable under different environmental conditions, such as arid regions, saline soils, or areas with extreme temperatures. Sensitivity to desiccation and the need for high dosages for large-scale applications further limit their practicality in some farming systems.^148^ Regulatory and knowledge gaps exist as there is a lack of standardized regulations and guidelines for the safe use, labeling and monitoring of bionanofertilizers, especially those containing engineered nanomaterials. Farmers and agricultural stakeholders often lack adequate knowledge and training regarding the correct use and potential risks associated with bionanofertilizers, hindering adoption.

Furthermore, the use of nanomaterials for delivering nutrients to crops is still in its early stages and requires further research, particularly concerning nanofertilizers for nitrogen and phosphorus.

The key concerns regarding nanoscale technology in agrochemicals include

• Scaling up the safe synthesis of sustainable nanomaterials for agriculture and developing nanoparticles for controlled nutrient release.

• Characterizing nanostructures to gain a comprehensive understanding of their nanoscale properties.

• Ensuring accurate delivery and absorption rates of nanomaterials while studying their metabolic fates.

• Creating nanoscale materials that serve dual purposes as fertilizers and pesticides for effective plant management.

• Exploring nano-bio interactions and the environmental impact of nanomaterials.

• Optimizing nanomaterials dosage for various crops through long-term studies.

• Understanding the interrelations between nanoparticle characteristics and their toxicity.

• Evaluating human and environmental exposure *via* food chains.

• Assessing potential environmental risks and developing mitigation strategies.

• Collaborating internationally to validate nanoproducts in real farming contexts.

• Establishing educational initiatives to address farmer concerns and enhance communication between scientists and agricultural communities.^108^

## 17 Conclusion

The integration of bionanofertilizers into agroecosystems presents transformative opportunities for enhancing agricultural productivity among environmentally sustainable practices slowly gaining traction. Large surface areas coupled with sluggish nutrient release render them suitable for modern agribusiness productivity and determined resistance against various stresses. Fertilizer use decreases while nutrient uptake increases, while losses dwindle under their effective management strategies. Seed coatings embedded with novel bionanofertilizers and bespoke formulations can reduce costs and mitigate environmental issues in production processes. Bionanofertilizers play crucial roles in sustainable agriculture nowadays underground. They release nutrients relatively slowly over 40–50 days and retain more nutrient content after field application than most synthetic fertilizers do. Bionanofertilizers boost crop yields and quality traits, with foliar application being vastly superior to soil application today. Research on the appropriate use of bionanofertilizers should be thoroughly carried out before their widespread commercialization happens rapidly across the country. Future studies should probe the bioavailability, toxicity and safety of diverse nanoparticles and bionanofertilizers and examine green-synthesized nano-biofertilizers to boost yields sustainably.

## Author contributions

Samukarani Swain: writing – preparation and editing of the original draft, formal analysis, resources, figures and tables. Lala Behari Sukla: conceptualization, visualization, supervision, review and editing. D. P. Krishna Samal: data curation, supervision, review and editing.

## Conflicts of interest

The authors declare that they have no known competing financial interests or personal relationships that could have appeared to influence the work reported in this paper.

## Data Availability

This manuscript is a review article, and no new experimental data, software, or code were generated or analyzed during this study. All information presented in this manuscript is derived from previously published literature, which has been appropriately cited within the article. For any other additional information, please contact the corresponding author at lalabeharisukla@soa.ac.in.
